# Complete genome sequence of *Escherichia coli* JCM 5491

**DOI:** 10.1128/mra.00853-24

**Published:** 2024-10-04

**Authors:** So Umekage

**Affiliations:** 1 Department of Food and Nutrition, Hakodate Junior College, Hakodate, Hokkaido, Japan; 2 Department of Food and Nutrition, Koriyama Women's University, Koriyama, Fukushima, Japan; The University of Arizona, Tucson, Arizona, USA

**Keywords:** *E. coli*, complete genome, JCM 5491

## Abstract

Here, I report the complete genome sequence of *Escherichia coli* JCM5491, which is widely used in microbiological experiments.

## ANNOUNCEMENT

*Escherichia coli* JCM 5491 is a synonymous strain of *E. coli* ATCC 25922 and is widely used for microbiological experiments and antimicrobial susceptibility tests (1, 2). Although the complete genome sequence of *E. coli* JCM 5491 has already been deposited in GenBank (GCA_000614625.1), the data have been suppressed because of numerous frameshifted proteins. Here, I present the complete sequencing obtained using both short- and long-read sequencing.

*E. coli* JCM5491 was obtained from the Japan Collection of Microorganisms. A single colony was isolated from the frozen glycerol stock by streaking on a Luria-Bertani (3) agar plate, and a single colony was selected and grown in shaking culture (passage number 2) for 13 hours at room temperature in Luria-Bertani broth. According to the manufacturer’s protocol, genomic DNA was obtained using the NucleoSpin Microbial DNA Kit (Macherey-Nagel, GmbH & Co. KG), and DNA fragments shorter than 25 kb were removed using a Short Read Eliminator Kit (Nippon Genetics). The DNA for the short- and long-read sequencing prepared from the same culture.

Long-read sequencing libraries were prepared using the SQK-RAD004 rapid sequencing kit and sequenced using MinION Mk1B (Oxford Nanopore Technologies) with an R9.4.1 flow cell (FLO-MIN106). Base calling and subsequent quality checks were performed using the CPU version of guppy version 6.1.0 (Oxford Nanopore Technologies) with 16 CPU threads per caller and NanoPlot version 1.40.0 (4). The summarized statistics were as follows: number of reads, 1,945,604; read length of *N*_50_, 7,152; and total number of bases, 8,214,837,069. Reads longer than 13 kb with quality over Q9, equivalent to approximately 200 times coverage, were assembled using Canu version 2.2 (5). The length of the resulting contig was 5,131,721 bp, with approximately 80× coverage. Single contig was obtained by manually removing the overlapping ends.

Short-read sequencing libraries were prepared using Illumina DNA Prep (M) Tagmentation (Illumina), and 151-bp paired-end reads were sequenced using NovaSeq6000 (Illumina) with the NovaSeq 6000 SP Reagent Kit version 1.5 (300 cycles). The total number of reads was 7,698,796 (2.30 Gbp in total). Adapter trimming and quality filtering were performed using fastp version 0.19.6 (6) with default settings except for trimming both 20 bp in front and 2 bp in the tail of the reads. Hybrid error correction, consisting of short-read mapping using BWA-MEM version 0.7.17-r1188 (7) against the long-read single contig and polishing using Pilon version 1.24 (8), was repeated four times until no base corrections were reported. The completeness, contamination, and strain heterogeneity were assessed using CheckM (9) (Table 1). An in-house Python script (rotate_string.py, DOI:10.5281/zenodo.13744203) was used to rotate *dnaA* to the top for data submission to the DNA Data Bank of Japan (DDBJ). The contig was annotated using the DDBJ Fast Annotation and Submission Tool (10). Table 1 represents summary data for the obtained complete genome sequence of *E. coli* JCM 5491. Default settings were used for tools except where otherwise stated.

**TABLE 1:** Statistics of assembled whole-genome sequence of *Escherichia coli* JCM 5491

	JCM 5491
Total length (bp)	5,131,718
Coverage	80×
Topology	circular
Completeness (%)	99.46
Contamination (%)	0.57
Heterogeneity (%)	0
*N*_50_ (bp)	5,131,718
Gap ratio (%)	0
GC content (%)	50.5
No. of CDSs	4,691
Coding ratio (%)	86.9
No. of rRNAs	22
No. of tRNAs	89
No. of CRISPRs	0

## Data Availability

The complete genome sequence of *E. coli* JCM 5491 was deposited in the DDBJ under the accession number AP035801. The raw reads (DRR545508 and DRR545509) were deposited to the DDBJ Sequence Read Archive (PRJDB17988).
